# Investigation on Indentation Cracking-Based Approaches for Residual Stress Evaluation

**DOI:** 10.3390/ma10040404

**Published:** 2017-04-12

**Authors:** Felix Rickhey, Karuppasamy Pandian Marimuthu, Hyungyil Lee

**Affiliations:** Department of Mechanical Engineering, Sogang University, Seoul 04107, Korea; felix@sogang.ac.kr (F.R.); pandian@sogang.ac.kr (K.P.M.)

**Keywords:** residual stress, indentation fracture, fracture toughness, extended finite element analysis

## Abstract

Vickers indentation fracture can be used to estimate equibiaxial residual stresses (RS) in brittle materials. Previous, conceptually-equal, analytical models were established on the assumptions that (i) the crack be of a semi-circular shape and (ii) that the shape not be affected by RS. A generalized analytical model that accounts for the crack shape and its change is presented. To assess these analytical models and to gain detailed insight into the crack evolution, an extended finite element (XFE) model is established. XFE analysis results show that the crack shape is generally not semi-circular and affected by RS and that tensile and compressive RS have different effects on the crack evolution. Parameter studies are performed to calibrate the generalized analytical model. Comparison of the results calculated by the analytical models with XFE results reveals the inaccuracy inherent in the previous analytical models, namely the neglect of (the change of) the crack aspect-ratio, in particular for tensile RS. Previous models should therefore be treated with caution and, if at all, used only for compressive RS. The generalized model, on the other hand, gives a more accurate description of the RS, but requires the crack depth.

## 1. Introduction

Residual stresses (RS) exist in many structures. They may have been induced intentionally (e.g., shot peening, chemical strengthening) or inevitably (e.g., cold working due to polishing, thermal treatment accompanied by phase transformation) and significantly affect fatigue life, corrosion or wear resistance, in a positive or negative way [1]. Tempering, for example, is a very effective means to improve the strength and contact damage resistance in glass ceramics. Here, compressive RS are introduced into the surface, thereby increasing the effective stress for damage initiation and propagation. RS can also enhance the mobility of charge carriers in semiconductor devices [2]. Hence, RS play a central role regarding the performance of brittle structures, and their determination has been of considerable interest [3,4].

Techniques for RS determination can be categorized into destructive and non-destructive techniques [5]. Destructive methods, such as hole-drilling, saw-cutting, curvature and layer removal [6], rely on the deformation due to the (partial) relief of RS upon removal of material. When employing non-destructive techniques (NDT), RS are usually inferred indirectly. NDT include ultra-sonic methods, micromagnetic methods, Raman spectroscopy, neutron or X-ray diffraction [7,8,9]. Many of these methods are however rather expensive or limited in their applicability.

A mechanical NDT, indentation, is a convenient, inexpensive and quick means for RS estimation and can be applied to ductile [10,11], as well as brittle materials [12]. Generally, RS support (tensile RS) or work against (compressive RS) the penetration of the material by the indenter, resulting in a downward (tensile RS) or upward shift (compressive RS) of the characteristic indentation force-indentation depth curve. Further, RS influence the pile-up/sink-in at the impression border. In the case of ductile materials, RS are determined directly from these RS-induced changes in resistance to indentation and pile-up/sink-in behavior. However, in the case of brittle materials, cracks may emanate from the corners of the impression or inside the material, depending on the indenter shape and material [13], and grow into a half penny-shaped crack. This method has been frequently used to measure the fracture toughness of ceramics and glasses. To evaluate RS, the sensitivity of the final crack dimensions to in-plane RS is made use of. This method is particularly advantageous for local subsurface RS determination and in cases where optical and other conventional methods such as fracturing in flexure are not employable. So far, spherical [14,15,16,17], conical [18], cube-corner [19,20], Vickers [15,21], Berkovich [22] and Knoop indenters [18] have been employed.

Tandon and Cook [23] and Koike et al. [24] investigated the differences in sharp indentation crack initiation and propagation between annealed, tempered and ion-exchange-strengthened glasses and noted that compressive RS yield a decrease in the propensity to initiation of radial and median cracks. Zeng and Rowcliffe [25] presented a Vickers indentation method to analyze the RS field around a highly-stressed region in a glass specimen (the RS field itself was generated by a Vickers indenter). Later, Kese and Rowcliffe [19] did the same with cube-corner indenters, but they assumed a semi-elliptical crack geometry, which is different from those assumed by Zeng and Rowcliffe [25] and Zeng et al. [26]; a new crack geometry factor was proposed based on a cube-corner indenter. However, the calculated RS were 2~4-times higher than those calculated by Zeng and Rowcliffe [25], Zeng et al. [26] and Peitl et al. [27]. Roberts et al. [16] and Bisrat and Roberts [28] exploited the shift in the threshold load for the propagation of a pre-existing surface crack to estimate RS. The Vickers indentation study by Peitl et al. [27] revealed that the radial-median cracks in the glass ceramic they employed are not of semi-circular, but of a semi-elliptical shape, which means that the appropriateness of assuming a semi-circular crack, as was done before, is questionable. To account for the departure from the semi-circular shape, they introduced a correction factor in the formulation of Zeng and Rowcliffe [27,29]. Rodríguez-López et al. [30] applied the method of Peitl et al. to evaluate the RS in laser-cladded glass-ceramic sealants on Crofer22APU steel. Therefore, the experimental part of this approach was limited to one material, i.e., glass ceramics (glass matrix).

Today, indentation fracture is, for example, used for determining RS in dental ceramics [31,32,33,34,35,36,37] or in shot-peened ceramics [38]. Thus far, indentation-based studies were limited to compressive RS and only a few materials (mainly glass). Previous analytical models were established on assumptions whose appropriateness has not been verified by experiments, which were qualitative rather than quantitative or numerical techniques. The goal here is therefore (i) to provide better insight into the influence of both compressive and tensile equibiaxial RS on subsurface crack evolution and final crack shape and size; (ii) how crack evolution is affected by relevant material properties and (iii) to scrutinize previous models and their underlying assumptions by comparison with a generalized analytical model and numerical results obtained by the extended finite element method (XFEM). To the authors’ knowledge, this is the first XFEM-based study on indentation of pre-stressed specimens.

## 2. Analytical Models

The difference in size of indenter-induced cracks in a stressed structure as compared with the RS-free equivalent can be explained by considering the RS field to be superimposing onto the indentation stress field [14]. The RS-induced change in the stress field affects crack initiation and propagation. Tensile RS increase the tensile wedging forces and thus cause earlier damage initiation and a larger final crack. Conversely, compressive RS result in restrained (or even totally suppressed) crack formation and thus a smaller final crack (or no crack) [15,39].

### 2.1. Shape Factor for a Semi-Elliptical Surface Crack Subject to Remote Tension

**Figure 1:**
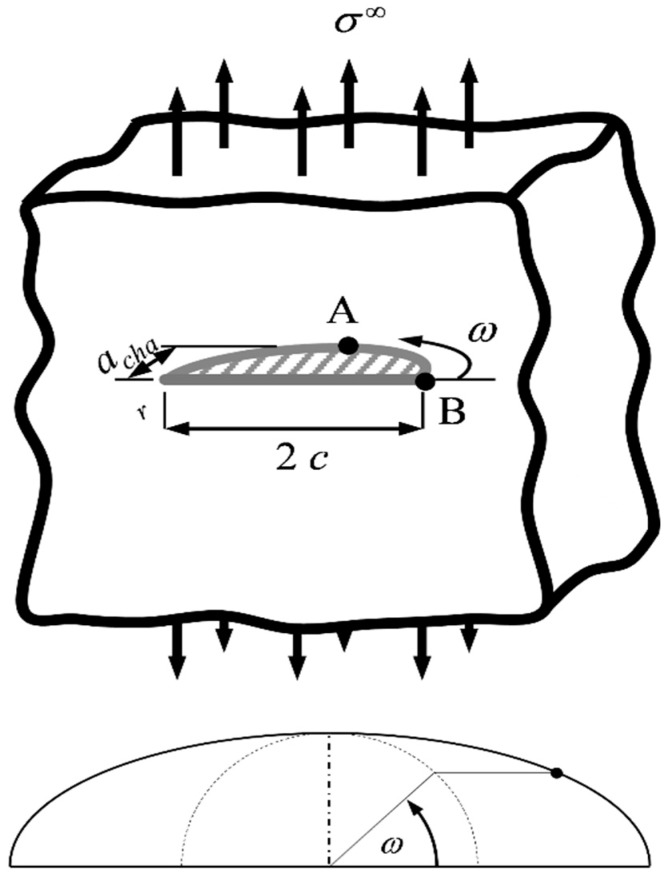
Semi-elliptical surface crack in a semi-infinite medium subject to remote tensile stress *σ*^∞^ (**top**) and the definition of the parametric angle (**bottom**) (following Anderson [41]).

**Figure 2:**
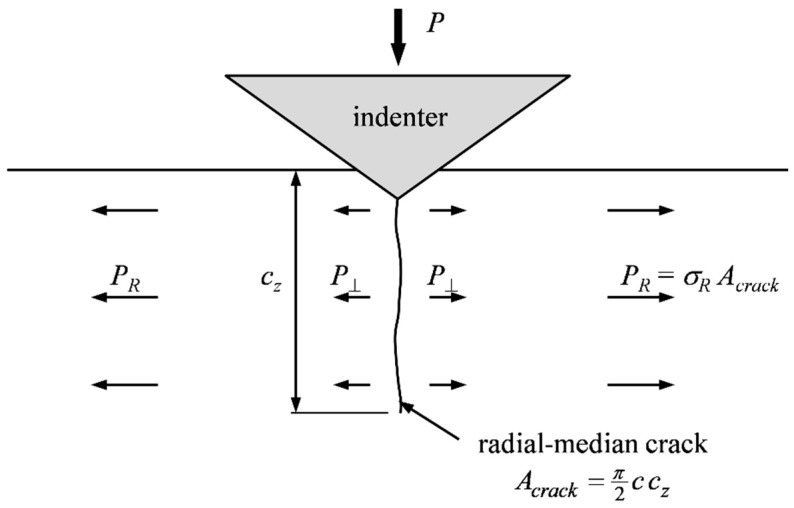
Residual force *P_R_* superimposes on wedging forces *P*_⏊_, both acting normal to the crack surface *A_crack_*.

**Figure 3:**
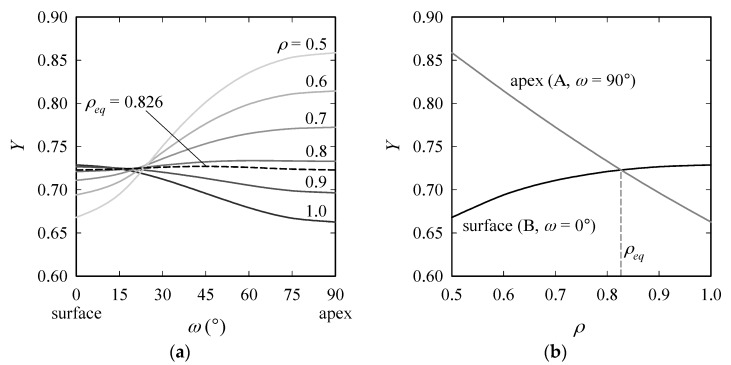
Change of shape factor *Y* with *ω* for (**a**) diverse *ρ* (Equation (2)) and (**b**) with *ρ* at Points A and B; for *ρ* = 0.826, *Y* has equal values at A and B.

Based on linear elastic fracture mechanics (LEFM), the mode I-stress intensity factor (SIF) for a crack subject to remote tensile stress *σ*^∞^ (Figure 1) can be expressed as:

(1) $$K=Y\sigma^{\infty }\sqrt{\pi a_{char}},$$

where *a_char_* is a characteristic crack dimension. The shape factor *Y* accounts for the crack configuration and the change of the SIF along the crack front. Here, we assume the crack to be semi-elliptical. *a_char_* = *c_z_* (crack depth), and the aspect ratio of the ellipse is *ρ* ≡ *c_z_*/*c*, where *c* is the length of the crack on the surface (Figure 1 and Figure 2). *Y* for a semi-elliptical surface crack with *ρ* ≤ 1 (in this study, *ρ* was always < 1) under mode I-loading conditions is [40]:

(2) $$Y\left(\rho ,\omega \right)=\frac{Mf_{\omega }g}{\sqrt{Q}},$$

(3) $$Q=1+1.464\rho^{1.65}\;\;;\;\;M=1.13-0.09\rho ,\;f_{\omega }={\left[\sin^{2}\omega +\rho^{2}\cos^{2}\omega \right]}^{1/4}\;\;;\;\;g=1+0.1\left(1-\sin^{2}\omega \right),$$

where *Q*, *M*, *f_ω_* and *g* are geometry factors and the parametric angle *ω* is 0° at the surface and 90° at the apex of the crack. The variation of *Y* along the crack front is plotted in Figure 3 (left) for diverse *ρ*. For *ρ* > *ρ_eq_* = 0.826, crack growth takes place at the surface (Point B), and for *ρ* < *ρ_eq_*, *K* becomes maximum at the apex (A). Regarding the change of *Y* at B with *ρ* in the range [0.7, 1], the maximum difference in *Y* is 2.5% (Figure 3, right).

### 2.2. Previous Approaches

Marshall and Lawn [42] and, shortly afterwards, Swain et al. [43] presented LEFM-based models, which are conceptually equal. The equibiaxial RS field was superimposed on the indentation stress field, as shown in Figure 2. Self-similarity requires that the indentation load *P* scale with the wedging force acting normal to the crack surfaces, *P*_⏊_. Since loading modes are consistent (mode I), the contributions to the total SIF *K_tot_* are additive; thus:

(4) $$K_{tot}=K_{ind}+K_{R},$$

where *K_ind_* and *K_R_* are the SIF due to the indentation stress field and the RS field, respectively. The crack was assumed to be of a semi-circular shape, i.e., *ρ* = 1, independent of material or RS. The SIF solution for a semi-circular surface crack in a semi-infinite medium subject to tensile stress *σ_R_* has the form:

(5) $$K_{R}=Y\left(1,\omega \right)\sigma_{R}\sqrt{\pi c},$$

which is maximum at the surface (*ω* = 0°). For indentation on an RS-free specimen (e.g., Lawn et al. [44]):

(6) $$K_{ind}=\chi \frac{P_{\max}}{c^{3/2}}\;\;,$$

where *χ* is a material- and indenter-dependent constant. At the crack tip during crack propagation, *K_tot_* = *K_c_*, where *K_c_* is the critical SIF (=fracture toughness). Inserting Equations (5) and (6) into Equation (4), Marshall and Lawn [42] arrived at the following expression for *σ_R_*:

(7) $$\sigma_{R}=\frac{\sqrt{\pi }\chi }{2m\sqrt{c}}\left[\frac{K_{c}}{\chi }-\frac{P_{\max}}{c^{3/2}}\right],$$

Correction *m* was set to unity, which means a neglect of free surface-effects and a homogeneous RS field. Indentations to diverse *P*_max_ were carried out on as-annealed soda-lime glass specimens (to obtain the material’s reference *K_c_*/*χ*) and tempered soda-lime glass specimens, and results gave an approximately linear relation between *P*/*c*^3/2^ and *c*^1/2^. Zeng and Rowcliffe [25] also applied Equation (7), but with *m* = *π*/2.

Swain et al. [43] suggested indenting RS-free and specimens subject to RS, so that equal final crack lengths *c_n_* are obtained. The compressive RS, *σ_R_*, was calculated from the load difference by:

(8) $$\sigma_{R}=\frac{\chi }{Y\sqrt{\pi }c_{n}^{2}}\left[P_{o\;\max}-P_{\max}\right],$$

where subscript ‘o’ denotes the (reference) RS-free case. *χ* is obtained from the indentation of the annealed (i.e., RS-free) specimen, and *P*_max_ is the maximum indentation load necessary to produce a crack of nominal length *c_n_* in the stressed material. Experiments on tempered and annealed soda-lime glass specimens verified the proportionality of *c_n_*^2^ and the load difference. While Swain et al. assumed *Y* = *π*^–1/2^, Chaudhri and Phillips [15] later accounted for the free surface and corrected the shape factor to *Y* = 1.16*π*^–1/2^.

When modifying the model by Swain et al. [43] so that it can be used for indentations to a nominal *P*_max_ (instead of *c_n_*), we can write the above analytical models in the following standard form:

(9) $$\sigma_{R}=\frac{\chi }{Y}\frac{P_{\max}}{\sqrt{\pi }c^{2}}\left[{\left(\frac{c}{c_{o}}\right)}^{3/2}-1\right];\;\;Y=\{\begin{array}{c} 0.56\;\;\;\;\;\;\;\mathrm{Swain}\;et\;al.\;\;\left[43\right]\\ 0.64\;\;\;\;\mathrm{Marshal}\;\mathrm{and}\;\mathrm{Lawn}\;\left[42\right]\\ 0.65\;\mathrm{Chaudhri}\;\mathrm{and}\;\mathrm{Phillips}\;\left[15\right]\\ 1\;\;\;\;\;\mathrm{Zeng}\;\mathrm{and}\;\mathrm{Rowcliffe}\;\;\left[25\right] \end{array},$$

The analytical models thus differ only by *Y*, which however ranges, depending on the source, between 0.56 and one. Two indentations, one on the RS-free and one on the specimen subject to RS, to equal *P*_max_, are required to get *c*/*c*_o_. For two indentations to equal *h*_max_ (as in our numerical analyses), Equation (9) becomes:

(10) $$\sigma_{R}=\frac{K_{c}}{Y\sqrt{\pi c}}\left[1-\frac{C}{C_{o}}{\left(\frac{c}{c_{o}}\right)}^{3/2}\right],$$

where *C*/*C*_o_ denotes the relative change of Kick’s law coefficient. Kick’s law denotes the linear relation between indentation load *P* and squared indentation depth *h*^2^, *P* = *C h*^2^, for indentation with symmetric sharp indenters on elastic-perfectly plastic materials [39]. Kick’s law coefficient *C* is then a material constant. Note that Kick’s law holds independent of RS or cracking. As stated before, Equation (9) assumes that *ρ* = 1 holds independent of material properties and indenter shape. However, *ρ* is expected to be material- and indenter-dependent (compare Rickhey et al. [45] for Knoop indentation of the RS-free specimen) and may, moreover, change under the influence of RS, so that *ρ*/*ρ*_o_ ≠ 1. The consequences of these assumptions will be scrutinized in Section 4.

### 2.3. Generalized Analytical Model

To assess the appropriateness of these assumptions, a generalized analytical model, similar to Peitl et al. [27], is suggested. The stresses in the elastic far-field are approximated by the Boussinesq solution for the stress distribution in an elastic half-space subject to a point load acting normal to the surface. The crack-driving circumferential stresses *σ_θ_* in a median plane (i.e., a plane normal to the specimen surface) are:

(11) $$\sigma_{\theta }\propto \frac{P_{⊥}}{\pi R^{2}}g_{\omega }\left(\omega \right),$$

where *P*_⏊_ is the force acting normal to the crack plane (Figure 2) and *g_ω_* is an angular function of *ω*, which is defined in Figure 1. At the surface (*R* = *c*, *ω* = 0°; Point B in Figure 1), where the crack propagates, the following relation exists for the SIF:

(12) $$K_{ind}\propto Y\frac{P_{⊥}}{\pi c^{2}}\sqrt{\pi c_{z}}=Y\sqrt{\rho }\frac{P_{⊥}}{\sqrt{\pi }c^{3/2}},$$

and the shape factor becomes:

(13) $$Y=\frac{1.243-0.1\rho }{\sqrt{1+1.464\rho^{1.65}}}\sqrt{\rho },$$

In accordance with self-similarity, the crack-driving force scales with the indentation load *P*_max_, i.e.,

(14) $$P_{⊥}=kP_{\max}\;,$$

where *k* is an indenter shape- and material-dependent scale factor. *k* is expected to be closely related to the residual field intensity factor *χ*. Plugging Equation (14) into Equation (12), we get:

(15) $$K_{ind}=Y\sqrt{\rho }\frac{kP_{\max}}{\sqrt{\pi }c^{3/2}},$$

The SIF contribution from the in-plane RS, which act as remote tensile stresses, i.e., *σ*^∞^ = *σ_R_*, is:

(16) $$K_{R}=Y\sqrt{\rho }\sigma_{R}\sqrt{\pi c},$$

During crack propagation *K* = *K_c_* must hold, that is:

(17) $$\sigma_{R}=0\to \;K_{tot}=K_{ind}=K_{c};\sigma_{R}\neq 0\to \;K_{tot}+K_{R}=K_{c},$$

Comparison of the equibiaxial RS case with the RS-free case gives:

(18) $$\sigma_{R}=\frac{k}{\sqrt{\pi }\chi }\frac{K_{c}}{\sqrt{\pi c}}\left[\frac{Y_{o}}{Y}\sqrt{\frac{\rho_{o}}{\rho }}-{\left(\frac{c}{c_{o}}\right)}^{3/2}\right]=\frac{kP_{\max}}{\pi c^{2}}\left[\frac{Y_{o}}{Y}\sqrt{\frac{\rho_{o}}{\rho }}{\left(\frac{c}{c_{o}}\right)}^{3/2}-1\right],$$

(It was eventually found that the variation of *Y*_o_/*Y* is very small, so that the term *Y*_o_/*Y* might as well be removed from Equations (18) and (19) without loss of accuracy. However, we do not remove it here for consistency.) In the case of indentations, to equal *h*_max_, Equation (18) changes to:

(19) $$\sigma_{R}=\frac{kK_{c}}{\pi \chi \sqrt{c}}\left[\frac{Y_{o}}{Y}\sqrt{\frac{\rho_{o}}{\rho }}-\frac{C}{C_{o}}{\left(\frac{c}{c_{o}}\right)}^{3/2}\right],$$

Note that for *ρ* = 1 and *ρ*/*ρ*_o_ = 1, Equation (18) reduces to:

(20) $$\sigma_{R}=\frac{kK_{c}}{\pi \chi \sqrt{c}}\left[1-{\left(\frac{c_{o}}{c}\right)}^{3/2}\right]=\frac{kP_{\max}}{\pi c^{2}}\left[{\left(\frac{c}{c_{o}}\right)}^{3/2}-1\right],$$

which is equal to Equation (9) with *k*/*χ* = *π*^1/2^/*Y*.

In summary, the assumptions common to both models, henceforth termed ‘simple’ (Equation (9) or (10)) and ‘generalized’ (Equation (18) or (19)), are that SIF are additive and that the crack shape can be sufficiently described by *ρ*. The ‘simple’ model further requires that the crack be semi-circular (*ρ* = 1) and that RS do not affect *ρ* (*ρ*/*ρ*_o_ = 1). The ‘generalized’ model, on the other hand, accounts for the material- and indenter-dependent crack aspect ratio and its possible change with RS.

The analytical models are not restricted to a particular sharp indenter. The non-interaction of radial-median cracks argues for Knoop indentation. However, since for general in-plane biaxial RS, the Vickers indenter will be advantageous (the non-equality of RS will be reflected by non-equal crack lengths in the median planes through the indenter diagonals, and provided sufficient sensitivity, only one indentation will be necessary), it is chosen here to make the study extendible to general biaxial RS.

## 3. FE Model and Imposition of Equibiaxial RS

**Figure 4:**
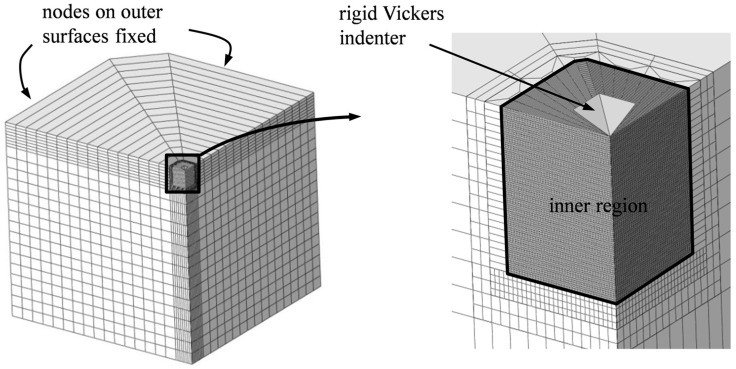
Quarter FE model for evaluation of residual stresses (RS) in brittle materials by Vickers indentation cracking.

Numerical analyses are performed with Abaqus/Standard. As the XFE model shown in Figure 4 is quite similar to the validated one in Rickhey et al. [45], this section is limited to essential information and to highlight differences. Owing to symmetry, modeling of one quarter specimen is sufficient. The model consists of ≈10^5^ eight-node brick elements [46]. All nodes on the outer surfaces of the model are fixed, so that RS can be introduced through the “initial conditions” option in Abaqus. Bottom nodes are fixed, as well. For simplicity, the indenter is restricted to movements in the *z*-direction and assumed rigid. The latter assumption is, however, not expected to influence the accuracy of the results to a degree that invalidates the approach, because the indenter’s elastic modulus is usually much higher than that of the material to be indented.

The material to be indented is assumed to exhibit elastic-perfectly plastic material behavior and to yield according to the von Mises yield condition (yield strength *σ_y_*). In fact, the compressive behavior of many brittle materials can be accurately described by this material model [47]. Damage is assumed to obey a bilinear continuum traction-separation law, governed by a damage-initiating stress threshold $\hat{\sigma }$ (=0.8 GPa) and the fracture energy *Γ*, which is tentatively set to 3.0 MPa µm. The dimensionless viscosity parameter, introduced to mitigate convergence problems associated with material softening, is set to *ζ* = 5 × 10 ^–5^. The role and choice of damage model parameters was discussed in detail in [45,48]. Friction between the indenter and specimen is considered with *µ* = 0.2. If not stated otherwise, all indentations were performed with a prescribed *h*_max_ = 1.5 µm.

## 4. FE Results and Observations for Equibiaxial RS

Parameter studies were carried out by varying material properties and equibiaxial RS to see whether and how equibiaxial RS affect cracking in general and *ρ*/*ρ*_o_ in particular. Finally, the RS calculated by the analytical models are compared with the RS input to the numerical analyses to discuss the importance of *ρ* and *ρ*/*ρ*_o_ regarding the accuracy of the results. First, we analyze the influence of RS on crack evolution for a reference material with properties close to polycrystalline silicon (*E* = 200 GPa, *ν* = 0.3, *σ_y_* = 5 GPa).

### 4.1. Observations Made for Reference Material

**Figure 5:**
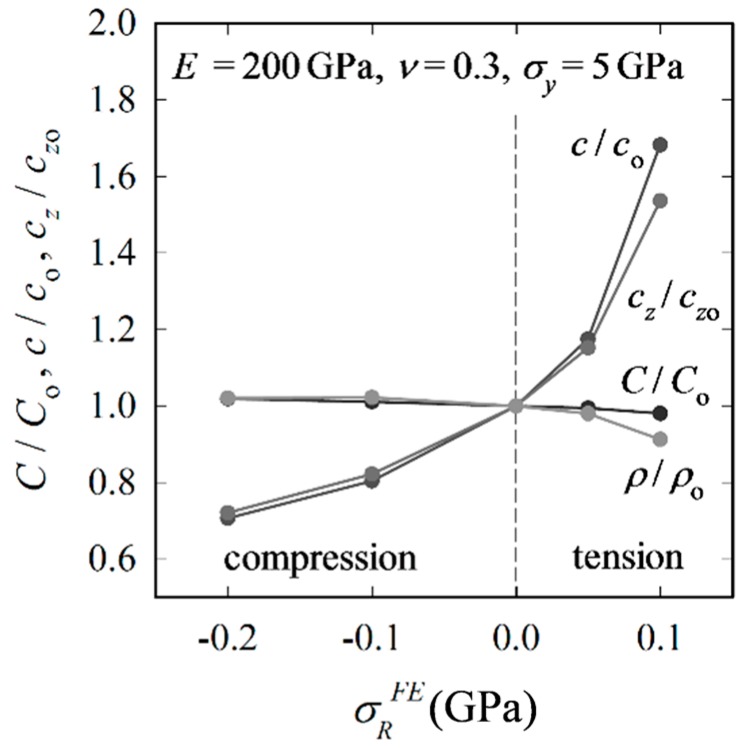
Relative change of Kick’s law coefficient *C*/*C*_o_, crack length *c*/*c*_o_, crack depth *c_z_*/*c_z_*_o_ and crack aspect-ratio *ρ*/*ρ*_o_ with *σ_R_^FE^* (FE input).

XFE results for the reference material reveal the following: The impression half-diagonal remains unaffected by RS, which agrees with Swain et al. [43]. Kick’s law (*P* ∝ *h*^2^) holds irrespective of RS and cracking. The decrease of *C* caused by cracking is negligible; as expected, tensile RS support the wedging process performed by the indenter so that *C* decreases with increasing RS (Figure 5). Compressive RS impede both the crack propagation in the depth direction during loading and the opening-up during unloading, whereas tensile RS have the opposite effect. The relative change in *c* and *c_z_* (normalized by ‘RS-free’ values *c*_o_ and *c_z_*_o_, respectively) is however more pronounced for tensile RS than for compressive RS.

**Figure 6:**
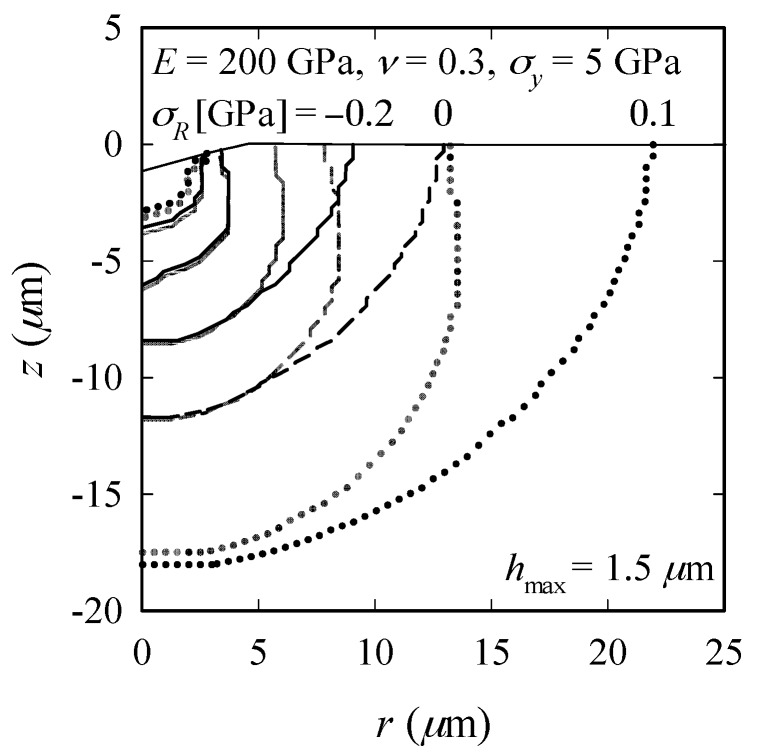
Influence of compressive and tensile RS on crack evolution at load reversal (semi-transparent lines) and after unloading.

Figure 6 demonstrates how RS influence the crack configurations at load reversal (*P* = *P*_max_) and after unloading (*P* = 0) for *σ_R_* = −0.2, 0 and 0.1 GPa. We observe that while for the RS-free and compressive RS cases, *c_z_* does not change during unloading, there is an increase in *c_z_* in the tensile RS case. Further, compressive RS do not cause an evident change in *ρ* (i.e., *ρ*/*ρ*_o_ ≈ 1), but *ρ* decreases with increasing tensile RS, indicating that the influence of tensile RS on radial-median cracking is fundamentally different from that of compressive RS. Note that a fundamental difference between tensile and compressive RS has also been observed in indentation of ductile materials (e.g., Sines and Carlson [49]; Suresh and Giannakopoulos [50]; Rickhey et al. [11]).

From sharp indentation of the RS-free specimen, we know that upon sufficient loading, *χ* becomes constant; the generated crack is then called well-developed. We now investigate the influence of RS on the residual field intensity at diverse *h*_max_. Let us for this purpose introduce an apparent residual field intensity coefficient *χ^app^* as follows:

(21) $$\chi^{app}\equiv \frac{K_{c}}{P_{\max}/c^{3/2}},$$

For the RS-free case, *χ^app^* = *χ*. Hence, with Equation (18), we get:

(22) $$\sigma_{R}=\frac{k}{\sqrt{\pi }\chi }\frac{K_{c}}{\sqrt{\pi c}}\left[\frac{Y_{o}}{Y}\sqrt{\frac{\rho_{o}}{\rho }}-\frac{\chi }{\chi^{app}}\right]=\frac{kP_{\max}}{\pi c^{2}}\left[\frac{Y_{o}}{Y}\sqrt{\frac{\rho_{o}}{\rho }}\frac{\chi^{app}}{\chi }-1\right],$$

for load-controlled indentations and, with Equation (19),

(23) $$\sigma_{R}=\frac{kK_{c}}{\pi \chi \sqrt{c}}\left[\frac{Y_{o}}{Y}\sqrt{\frac{\rho_{o}}{\rho }}-\frac{C}{C_{o}}\frac{\chi }{\chi^{app}}\right],$$

for depth-controlled indentations (note that *K_c_* is a material property and as such independent of RS). In a similar fashion, we introduce:

(24) $$\chi_{z}^{app}\equiv \frac{K_{c}}{P_{\max}/c_{z}^{3/2}}\;,$$

**Figure 7:**
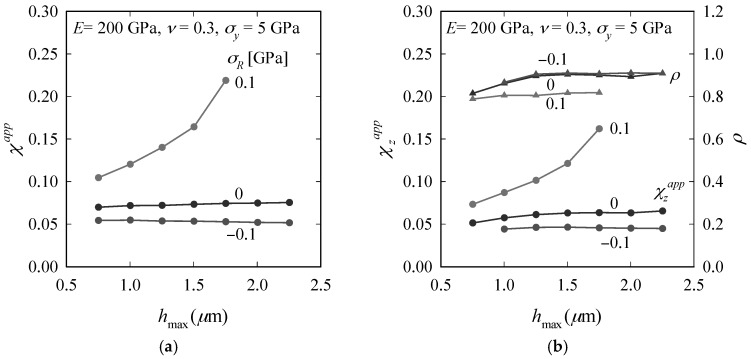
Change of *χ^app^* (**a**); *χ_z_^app^* and *ρ* (**b**) with *h*_max_ for *σ_R_* = 0.1, 0 and −0.1 GPa.

When plotting *χ^app^* obtained from indentations to *h*_max_ = [0.75, 2.25] µm, Figure 7 further demonstrates the different nature of compressive and tensile RS. (Note that some combinations of *h*_max_ and *σ_R_* could not be considered because of the limited size of the inner region (high *h*_max_, high *σ_R_*) or because of suppressed radial-median crack formation (low *h*_max_, low *σ_R_*). The size of the inner region is limited by computational costs, which are determined by *h*_max_, the chosen element size and $\hat{\sigma }$ [45].) *χ^app^* and *χ_z_^app^* remain constant for compressive RS (as known from the RS-free case), but increase clearly with *h*_max_ for tensile RS; *ρ* [= (*χ^app^*/*χ_z_^app^*)^2/3^] is independent of RS for compressive RS, but decreases with increasing RS for tensile RS.

**Figure 8:**
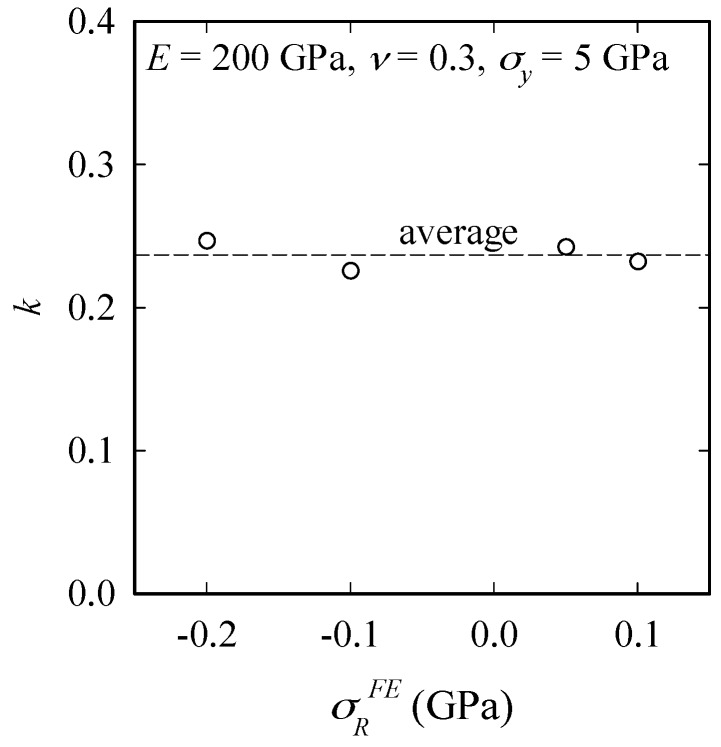
Coefficient *k* in Equation (19) vs. *σ_R_*.

The coefficient *k* in Equation (19) is found by calculating *σ_R_^FE^*/(*σ_R_^Eq^*/*k*), where *σ_R_^FE^* is the RS imposed in the FE analysis and *σ_R_^Eq^* is the RS calculated by Equation (19). Plotting *k* from Equation (19) against RS (Figure 8), we see that *k* is approximately constant for the reference material (≈0.24). This will be discussed in more detail in the next section.

### 4.2. Influence of Material Properties

To determine how the relation between *k* and *χ* is influenced by material properties, we performed parameter studies varying *E*, *ν* and *σ_y_*, so that a large range of brittle materials is covered (Table 1). It is to be noted that the pair (*E*, *σ_y_*) represents the usually given pair (*E*, *H*); the conversion, which is necessary for application in numerical analysis, is described in [45]. Indentation cracking tests were simulated with *σ_R_* = {–0.2, –0.1, 0, 0.05, 0.1, 0.15} GPa. Due to the high sensitivity to RS for low modulus materials (as we will see later), *σ_R_* = 0.15 GPa was applied only to materials with *E* = 600 GPa. RS lower than –0.2 GPa were not considered because radial-median cracks could not be generated (not even when increasing *h*_max_ to 3.0 µm). Higher RS were not imposed because they would have required a very large inner region (Figure 4) and thus significantly higher computational effort.

**Figure 9:**
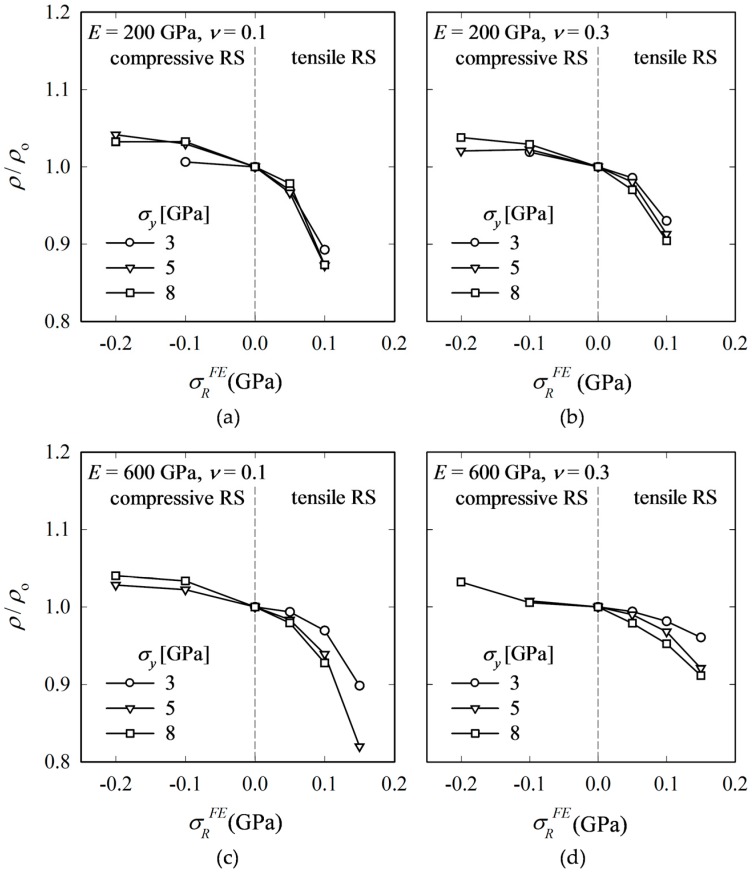
Normalized crack ratio *ρ*/*ρ*_o_ for all combinations of *E* = {200, 600} GPa, *ν* = {0.1, 0.3} and *σ_y_* = {3, 5, 8} GPa (note that not for all materials have radial-median cracks formed).

Figure 9 shows the change of *ρ*/*ρ*_o_ for four materials. *ρ* is found to be affected by compressive RS to a relatively low degree, which means that regarding *ρ*, a material-dependent, but RS independent constant may be acceptable. However, in the case of tensile RS, *ρ* becomes quite sensitive and decreases rapidly with increasing RS. The decrease of *ρ*/*ρ*_o_ is tantamount to a more pronounced opening-up of the crack at the surface (B).

Further, independent of material, tensile RS cause the crack to grow in the depth direction during unloading. With increasing RS, the crack shape approaches that of the equilibrium ellipse (*ρ_eq_*), whereupon the crack grows also in the depth direction during unloading because the SIF reaches *K_c_* at both A and B. For sufficiently high tensile RS, *ρ* is expected to be that of the equilibrium ellipse. For low RS, on the other hand, *c_z_* remains constant (only influenced by elastic unloading), which is consistent with theory (because *ρ* < *ρ_eq_*).

**Figure 10:**
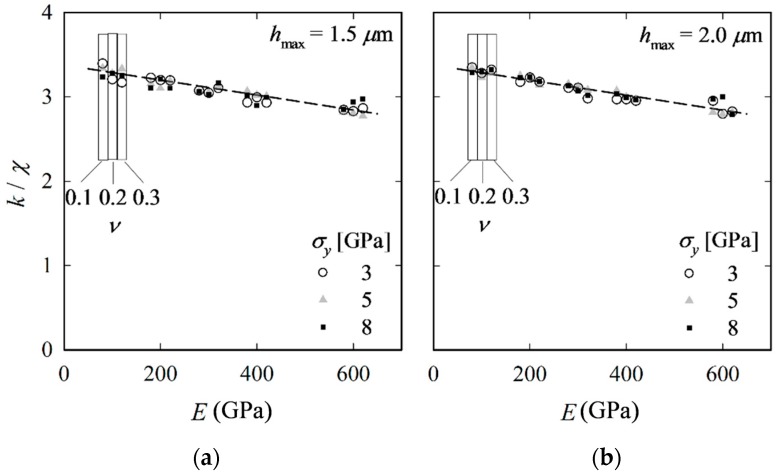
Mean average values of *k*/*χ* over the whole range of equibiaxial RS states vs. *E*; *h*_max_ = 1.5 (**a**) and 2.0 µm (**b**); data points for *ν* = 0.1 and 0.3 are plotted slightly left and right, respectively, of their real location for better visibility.

As mentioned in Section 2, the coefficient *k* is expected to be closely related to *χ*. Plotting results for *k*/*χ*, we find that, despite some scatter, *k*/*χ* shows no systematic dependence on *σ_y_* and *ν*, yet a low linear dependence on *E* (Figure 10). To show that results are independent of *h*_max_, the procedure is performed again, but now with *h*_max_ = 2.0 µm (instead of 1.5 µm), which, according to Kick’s law, means a 1.8-times increase in *P*_max_. The results in Figure 10 clearly show that *k*/*χ* is not affected by *h*_max_ (or *P*_max_). Based on the FE results for both *h*_max_, *k*/*χ* can be related to *E* through:

(25) $$\frac{k}{\chi }=a+bE,$$

where *a* = 3.37 and *b* = −9.07 × 10^−4^ GPa^−1^. The corresponding curve is shown by the dashed lines in Figure 10. The scatter may be explained, at least partly, by the inaccuracies associated with obtaining *c* and *c_z_* through interpolation.

**Figure 11:**
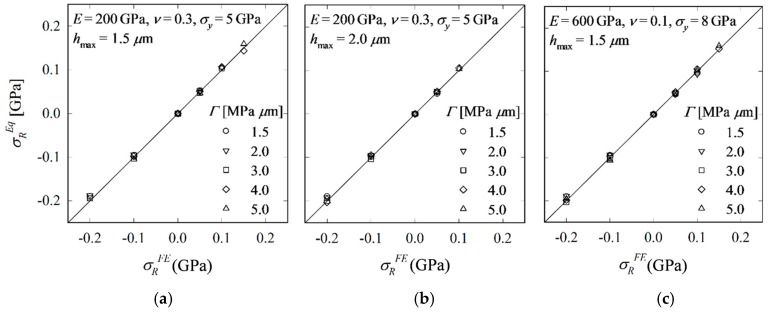
Comparison of RS calculated by Equation (19) (*σ_R_^Eq^*) with FE input values (*σ_R_^FE^*) for materials with different *Γ*; *E* = 200 GPa, *ν* = 0.3, *σ_y_* = 5 GPa; *h*_max_ = 1.5 (**a**) and 2.0 µm (**b**) and *E* = 600 GPa, *ν* = 0.1, *σ_y_* = 8 GPa, *h*_max_ = 1.5 µm (**c**).

To show that the ‘generalized’ model is further independent of *Γ*, *Γ* is varied in the range [1.5, 5.0] MPa µm (to reduce computational expense, not all combinations are analyzed). Two materials are considered: the reference material (*h*_max_ = 1.5 and 2.0 µm) and the material with *E* = 600 GPa, *ν* = 0.1, *σ_y_* = 8 GPa (*h*_max_ = 1.5 µm). Plugging *k*/*χ* from Equation (25) into Equation (19), RS (*σ_R_^Eq^*) are calculated and compared with the FE input values (*σ_R_^FE^*). The results plotted in Figure 11 reveal that deviations of *σ_R_^Eq^* from *σ_R_^FE^* are not systematic and are in the range obtained for *Γ* = 3 MPa µm. Equation (19) is thus independent of *h*_max_ and *Γ*.

### 4.3. Comparison of Analytical Models and Conclusion 

**Figure 12:**
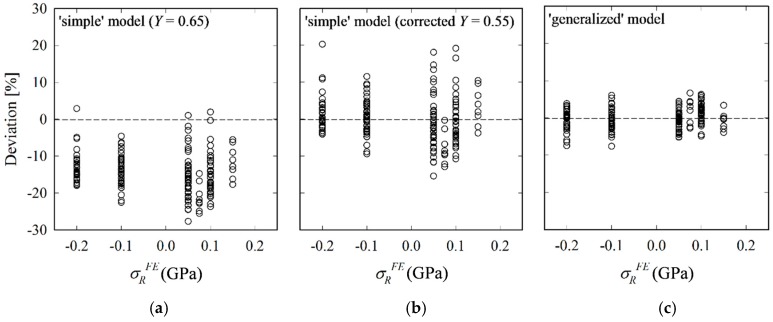
Deviations (=(*σ_R_^Eq^* – *σ_R_^FE^*)/*σ_R_^FE^*) of results calculated by ‘simple’ (**a**); corrected ‘simple’ (**b**) and ‘generalized’ analytical models (**c**) from FE input RS.

The RS calculated by the simple and generalized analytical models are compared with FE input values. As can be seen from Figure 12, the ‘simple’ model with *Y* = 0.65 underestimates RS systematically with errors close to 30%. The error is particularly large for tensile RS. When reducing the average deviation to zero by applying *Y* = 0.55, the error, albeit not anymore systematic, still amounts to up to 20%. Results obtained by the ‘generalized’ model are in a much better agreement with FE results. Maximum and average errors are reduced from 20% and 5% to 8% and 2%, respectively.

The improved accuracy hints at the importance of the crack aspect-ratio *ρ* and its change with RS, in particular for tensile RS. We conclude that for the case of compressive RS, *ρ* and its change caused by RS may be disregarded at the expense of some loss of accuracy. The ‘simple’ model with a corrected *Y* of 0.55 can thus be used in cases where (i) a quick evaluation of compressive equibiaxial RS is needed and (ii) the crack depth is unknown or difficult to obtain. However, one should be careful when applying it to the estimation of tensile RS because the results become inaccurate owing to the significant deviation of *ρ* from *ρ*_o_, as shown in Figure 9. The ‘generalized’ model, on the other hand, is more accurate, but only applicable when the crack depth can be measured, e.g., by focused ion beam (FIB) tomography [51].

## 5. Conclusions

The high sensitivity of crack formation to RS makes indentation fracture a powerful non-destructive tool for the evaluation of local subsurface RS. Previous analytical models presupposed (i) a semi-circular crack shape, i.e., *ρ* = 1; and (ii) that the ratio *ρ*/*ρ*_o_ does not change in the presence of RS, i.e., *ρ*/*ρ*_o_ = 1. However, results revealed that *ρ* (including *ρ*_o_) is smaller than one and that *ρ*/*ρ*_o_ clearly decreases with increasing tensile RS. Further, the influence of tensile RS on the crack evolution is notably different from that of compressive RS. While for compressive RS, the change of *ρ*/*ρ*_o_ is rather small and may be neglected, it should be considered in the case of tensile RS, which demonstrates the weakness of the ‘simple’ model. The proposed ‘generalized’ model, which accounts for (the change of) the crack aspect ratio *ρ* predicted RS much more accurately than the previous models, indicating the importance of (the change of) *ρ*, in particular for the tensile RS case. It is, however, to be noted that indentation cracking is not applicable in the case of very high compressive RS because the RS inhibit the development of the median crack into a well-developed radial-median crack. Higher tensile RS were not treated here because of the high sensitivity of the final crack size to even small increases in RS. We intend to tackle this issue by experiment in the near future.

Despite the simplicity of the previous approach (especially the independence of *c_z_*), it contains assumptions that lead to inaccuracies and should therefore be used, if at all, only for compressive RS. In connection with the previous model, a shape factor of *Y* = 0.55 was found to give an acceptable prediction of compressive RS. The model proposed here is recommended in the case of tensile RS and where the crack depth can be measured. 

## Figures and Tables

**Table 1:** Range of material properties applied in the parametric numerical simulations.

Material Properties	Values
*E* (GPa)	100, 200, 300, 400, 600
*ν*	0.1, 0.2, 0.3
*σ_y_* (GPa)	3, 5, 8
